# The role of simulation in the development of endovascular surgical skills

**DOI:** 10.1007/s40037-015-0250-4

**Published:** 2016-01-21

**Authors:** Craig Iain Nesbitt, Nikhil Birdi, Sebastian Mafeld, Gerrard Stansby

**Affiliations:** Northern Vascular Centre, Freeman Hospital, Newcastle Upon Tyne, England; Department of Radiology, Freeman Hospital, Newcastle Upon Tyne, England

**Keywords:** Endovascular skills, Medical simulation, Medical education

## Abstract

Endovascular trainees in the National Health Service still largely rely on the apprentice-apprenticeship model from the late 19th century. As the scope for endovascular therapy increases, due to the rapid innovation, evolution and refinement of technology, so too do patients’ therapeutic options. This climate has also opened the door for more novel training adjuncts, to address the gaps that exist in our current endovascular training curriculum. The aim of this paper is to present a succinct overview of endovascular simulation, synthesizing the trials and research behind this rapidly evolving training as well as highlighting areas where further research is required. The authors searched MEDLINE and EMBASE for relevant manuscripts on all aspects of endovascular simulation training. A comprehensive Google search was also undertaken to look for any relevant information on endovascular training courses available and any unpublished work that had been presented at relevant scientific meetings. Papers were categorized into the four models: synthetic, animal, virtual reality and human cadaver, and separate searches for evidence of skill transfer were also undertaken. Authors of novel research projects were contacted for further details of unpublished work and permission granted to report such findings in this manuscript.

## Essentials

- The field of endovascular intervention is rapidly evolving.
- Endovascular simulators provide a safe and efficacious platform for trainees to hone their basic skills.
- New advances in technology mean simulators can continue to provide an important training adjunct for even the most experienced practitioners. However, benefit gained is dependent on the choice of simulation model.
- Virtual reality simulation is the most promising, but cost remains a prohibitive factor.

## Introduction

Surgical practice is changing rapidly as technology continues to improve. The introduction of minimally invasive surgery has changed specialities such as gynaecology, urology and general surgery considerably.

Endovascular treatment options offer both reduced morbidity and mortality when compared with their equivalent open procedure options [1] and play a crucial diagnostic and therapeutic role in almost all branches of surgery.

The increase in therapeutic endovascular treatment options has also led to a need to tackle the issue of training in endovascular skills for the practitioners of the future, not the least because endovascular surgery requires a different set of skills, not only technical but cognitive as well, when compared with open surgery [2]. Indeed operating in a three-dimensional field from a two-dimensional view altered haptics and emphasis on hand-eye coordination are all challenging skills to master [3, 4].

Endovascular trainees in the National Health Service still largely rely on the apprentice-apprenticeship model from the late 19th century with the associated medico-legal and ethical ramifications. The introduction of minimally invasive arterial diagnostic techniques (duplex ultrasonography and magnetic resonance angiography) has diminished trainees access to diagnostic angiography, previously considered the baseline training procedure of the novice practitioner. Furthermore, as the scope for endovascular therapy increases, due to the rapid innovation, evolution and refinement of technology, so too do patients therapeutic options. Those previously unsuitable for open complex vascular procedures are increasingly brought to the endovascular specialist’s table. A steadily ageing population with ever more complex pathology are less suitable for junior practitioners and subsequently endovascular therapy tends to be a consultant-led practice.

This climate has also opened the door for more novel training adjuncts, to address the gaps that exist in our current endovascular training curriculum. The Chief Medical Officer acknowledged in his 2008 annual report that simulation affords a crucial role in safer patient care, and went on to recommend simulation-based training to become fully integrated and funded within the training curricula of surgeons at all training stages.

The aim of this paper is to present a succinct overview of endovascular simulation, synthesizing the trials and research behind this rapidly evolving training as well as highlighting areas where further research is required.

## Background

### History of medical simulation

‘Harvey’, an animated mannequin, is widely acknowledged to be one of the first developed medical simulators and was created as a cardiology simulator capable of task training using a computer-enhanced mannequin model [5]. Many other specialities have adopted simulation into their training curricula.

Despite improvements in simulator technology and an ever increasing popularity amongst trainees and trainers alike, conclusive evidence remains poor as to their exact benefit. A 2004 systematic review failed to demonstrate a firm advantage from expensive high-fidelity surgical simulators [6]. It is proposed that simulation is merely an adjunct, and not a replacement for clinical experience [7], which remains the gold standard. In general terms, medical simulation methods can be categorized into six levels, from level zero, which includes written scenarios to stimulate discussion, through to level six where interactive simulators provide a realistic experience [8].

Yet few can dismiss the distinct advantages that simulators confer. For trainees—an opportunity to make mistakes in a safe environment, witness the consequences of these mistakes, and learn from them. For trainers—a chance to examine the competence of trainees without putting patients at risk.

## Methods

The authors searched MEDLINE and EMBASE for relevant manuscripts on all aspects of endovascular simulation training. A comprehensive Google search was also undertaken to look for any relevant information on endovascular training courses available and any unpublished work that had been presented at relevant scientific meetings. Papers were categorized into the four models; synthetic, animal, virtual reality and human cadaver and separate searches for evidence of skill transfer were also undertaken. Authors of novel research projects were contacted for further details of unpublished work and permission granted to report such findings in this manuscript.

## Results

### Simulation and endovascular skills

Clinicians and trainers often refer to the ‘learning curve’ of trainees acquiring technical skills referring to the time it takes or the number of attempts required before the learner achieves safe independent competence [9]. A surgeon’s productivity and ‘learning curves’ are highly specific to that individual. It is recognized that during the early part of that curve, most mistakes and errors will be made by the novice operator [9]. This understanding means training on patients at this stage could increase their risk of morbidity, and therefore seems unethical.

Gallagher et al. reviewed the surgical education, human factor, and psychology literature in relation to the integration of virtual reality training into the training programme for minimally invasive surgery [10]. They concluded that simulation is efficacious in positively influencing the early part of the learning curve and this results in safer practice and more economic use of the operating theatre. However, virtual reality must be fully integrated into a well thought out education and training programme for it to successfully improve practitioners technical skills [10].

Endovascular practitioners exhibit this procedure-related learning curve at both novice and expert standard, and hence their patients are at risk during this phase of learning. A study of 200 consecutive coronary artery stenting procedures demonstrated a clear procedure-related learning curve and improved performance with fewer errors by practitioners of greater experience [11].

Acknowledging the patient-related safety advantages of operator experience, there are a number of trials that demonstrate the improved performance of endovascular practitioners following simulator training. Concentrating on renal intervention, Aggarwal et al. [12] trained 20 novice endovascular practitioners to perform angioplasty and stenting of the left renal artery, on a portable high-fidelity endovascular simulator. After only three repetitions all candidates demonstrated more efficient use of intravenous contrast and quicker procedure times [12]. Boyle et al. [13] constructed their trial to assess the importance of feedback in endovascular technical skills acquisition. They demonstrated significant performance improvements and fewer errors in all of their candidates performing a renal artery angioplasty and stenting following six repetitions on the simulator irrespective of their feedback. However greater improvements were seen in the feedback groups [13].

The efficacy of simulator training is also true for distal occlusive disease. Dawson et al. [14] demonstrated improvements in time, fluoroscopy use, volume of injected contrast and management of complications in nine candidates performing iliac angioplasty and stenting following 8 h of training on a high-fidelity simulation model. Similarly following didactic endovascular skills training Chaer et al. [15] randomized ten of their 20 recruited candidates to receive additional simulator training. All candidates then performed ilio-femoral angioplasty and stenting. Candidates’ performances were videoed and scored by blinded, expert assessors, using a validated scoring tool. Simulator trained candidates demonstrated improved measures of performance.

Simulation training is not necessarily appropriate for all practitioners. Dayal et al. [16] demonstrated that following simulator training for coronary artery stenting, experts (candidates who had performed more than 300 endovascular procedures) showed no significant improvement in performance. Therefore, the greatest training benefit from simulators is seen in inexperienced trainees who can develop and hone their basic guide-wire and catheter skills at the beginning of the ‘learning curve’, and these will become automated before they perform procedures in real patients [10].

### Simulation models for endovascular training

Simulated models for endovascular training can be divided into four broad categories: synthetic, animal, virtual reality and human cadaver.

### Synthetic

Synthetic models are a simple and cost-effective means of training. Models range from basic low-fidelity plastic models, to high-fidelity systems that incorporate pulsatile flow and fluoroscopic imaging [2]. Generally synthetic models are simple to use and set up and do not require X-ray radiation. Furthermore, due to their transportability, these models can be used outside the clinical environment, which makes these models more accessible.

A study using a cerebral aneurysm silicone model for neuro-endovascular intervention demonstrated frictional resistance and inability of devices to pass through curves in vessel walls [17]. Also, synthetic models fail to reproduce the dynamic behaviour of the arterial system, and they are unable to provide realistic simulated tasks for advanced procedures such as carotid artery stenting. Despite literature supporting the efficacy of low-fidelity training in minimally invasive surgery [18], there is no such evidence to support the validity in an endovascular setting.

A UK group has recently launched the world’s first fully three-dimensional printed bench top endovascular training model. Working with one of Europe’s leading medicinal three-dimensional printing companies (Materialise™) the inventors model includes a full scale, patent and transparent aorta with associated branches from the aortic root to the superficial femoral arteries enabling trainees to access vessels using genuine endovascular equipment. The group are proposing to publish results from an early trial of face validity in 2016.

### Animal

Animal models offer superior face validity compared with synthetic models for endovascular training [19]. A full spectrum of procedures in a fully functioning arterial tree can be performed, and realistic endovascular access using a percutaneous or surgical cut-down technique is feasible. Even the lack of natural pathology has also been overcome with artificial induction of both occlusive and aneurysmal disease through iatrogenically injuring vessel endothelium [20] and suturing constricting prosthetic patches around surgically exposed vessels [21]. The Porcine Transfer Study [22] showed significantly improved performance parameters in novices undertaking an iliac stenting procedure after training on a porcine model.

Despite enhanced fidelity and the proven validity of the model, the anatomy of animals differs from that of human subjects and vessels are much smaller, thus limiting access and device insertion. Cows or large apes would overcome this size discrepancy but they are too expensive and rarely used [23].

Further limitations of animal models include the logistics of setting up the training facility, including trained staff, radiographers, anaesthetists and an operating suite [19]. Animals can only be used for a single training session, which adds to the expense. In fact a detailed economic analysis revealed a difference of $ 1200 per candidate when training with a porcine model was compared with virtual reality simulation [24]. The home office have granted the first licence for live animal training in endovascular skills at a training facility in Northwick Park Institute for Medical Research. We eagerly await the results of any subsequent research and feedback as time and training in this unique centre progress.

### Virtual reality

Virtual reality is a communication interface based on interactive three-dimensional visualization allowing the trainee to interact and integrate different sensory inputs that simulate important aspects of real-world experience [25]. Endovascular virtual reality systems use these computer-generated images of the human vasculature to allow trainers the ability to interact with the model using an interface device [26]. A generic reusable instrument is inserted into the simulator model and the active tip is recognized by the machine, and displayed on the fluoroscopy screen in whatever form that has been pre-selected by the learner. In this manner, wires, catheters, stents, angioplasty balloons and coils can all be inserted in this simulated fashion.

Most high-fidelity models allow the trainer the option of adjusting the simulated C-arm, road mapping and cine-loop recording. Modules include iliac, aortic, renal, carotid, thoracic, coronary and neuro-intervention. Each contains graded scenarios from easy to difficult cases, introduced with a clinical monologue. Many simulators include real-time cardiovascular monitoring, which is displayed alongside the simulated fluoroscopy screen. Models are able to record performances, to enable trainers to assess candidates who can train at their convenience and receive feedback at the convenience of the trainer. Models can also provide post-procedure feedback on a number of different qualitative parameters. These include total procedure and fluoroscopy time, volume of contrast agent used, residual stenosis, accuracy of stent graft placement, and lesion coverage [3].

There are disadvantages to virtual reality models. Units cost in excess of $ 100,000 with added maintenance and recalibration costs, which can be considerable as these models are prone to technical failure. However, virtual reality models are well placed to offer endovascular skills training, offering a perfect medium for simulating the two-dimensional fluoroscopic imagine. There are no ethical issues related to their use and procedures can be repeated indefinitely. They allow the more novice trainee an opportunity to hone their guide-wire handling skills, and more expert practitioners a chance to rehearse new procedures in a safe environment prior to operating on patients. There is also great interest in the role of virtual reality as a model for objectively demonstrating procedural competence as part of a credentialing process [2].

The most recent advances include the option of downloading real patient images into the virtual reality machine. Models can then simulate that very case allowing practitioners an opportunity to rehearse challenging cases prior to the real performance. Some training facilities have simulated suites capable of performing procedures with a full theatre team. The results of a recent face validity study strongly support the use of such comprehensive simulation, demonstrating that once immersed in this authentic multidisciplinary simulated environment, trainees learn operative technical, procedural and management skills [27].

### Human cadaver

As an adjunct for medical training, human cadavers have played an integral role for many years. Yet current undergraduate trainees perform less cadaveric dissection in favour of fixed prosection specimens and synthetic models. Any enhanced benefit from cadavers could be offset by the fact that few medical students will go into a surgical career [28]. For this reason Reed et al. concluded that the anatomy lab is not an effective undergraduate educational environment [28].

However since the 2004 Human Tissue Act, doctors in the UK have been allowed to practice surgical procedures on cadavers for training and research purposes. This has led to a rising number of human cadaveric-based workshops in higher surgical training. Cadavers offer the perfect training compromise, offsetting the added risks of operating on human subjects, the ethical and legal implications of animals and the improved fidelity of synthetic or simulated models.

Human cadavers have proved useful for training in both open and minimally invasive surgical techniques. Training courses using such models are highly satisfactory for trainers and trainees alike [29].

The value of practising on human tissue, using real surgical instruments, offers a unique environment that perfectly simulates the surgical anatomical understanding and visuospatial awareness required when operating on live cases.

However, the downsides include that of cost and logistics. Dedicated training facilities with a Human Tissue Authority license are costly to set up, run and maintain. Using cadavers for multiple specialities is one way of keep down costs, for example, the same cadaver can undergo an orthopaedic course for lower limb prosthesis, a colorectal course for laparoscopic bowel resection, and an ear nose and throat course for septo-rhinoplasty. However, despite these cost-saving strategies, the transport, storage and the eventual disposal of cadavers that have been donated as anatomical gifts still make it a relatively expensive method for training.

Operator competence is usually measured in terms of technical proficiency. In an endovascular context this involves both technical and nontechnical skills. There are a number of theories in the literature that postulate how one learns a technical skill. All models of skill acquisition acknowledge the importance of intense, deliberate repetitive practice when mastering a technical skill.

### Fitts and Posner’s theory

This theory of motor skill acquisition follows three distinct stages [30]. The earliest *cognitive stage* is during which the trainee intellectualizes the task, getting to grips with the various steps and stages of the skill. In an endovascular setting this will involve familiarization with the various wires and catheters and learning to work with fluoroscopy. Progression to the second *integrative stage* comes with practice, and performance is seen to flow with fewer interruptions, but the trainee will still be observed thinking about how to progress with the next procedural step. The final *autonomous stage* is demonstrated with fluid uninterrupted performance, the trainee is no longer concerned with thinking of the next step in the task, but refining the finer elements of the procedure [31].

### Kopta’s theory

Similar to Fitts and Posner, Kopta believed in a three-phase progression towards skill acquisition. Improvement requires practice and feedback before the final autonomous phase, where the performer operates without cognitive input [32].

### Schmidt’s schema theory

Schmidt’s theory is based on how our motor skill acquisition develops. Every time a trainee performs a movement four pieces of information are gathered: the initial starting point information, aspects of the motor action itself, the success or failure of that action and finally the sensory consequences. In essence he believes that improvement requires practice in a wide variety of situations and encountering errors is equally important. Practice that lacks variety will not provide the learner with sufficient information and the learner will not fully comprehend the relationship between the manoeuver outcome and their control of the movement parameters [33].

### Ericsson’s model

Ericsson’s model focused more on the concept of expert performance [34]. He defines surgical experts as those with consistently better outcomes than non-experts. Attaining such a status is the result of dedicated and deliberate practice. Ericsson believed mornings were the best time to practice, as this was when the ability to perform complex tasks was highest. Although emphasis today has moved away from just sheer volume as a marker of competence, literature does exist to support the theory that operative volume and clinical outcome are related [34]. Ericsson used this to postulate that in fact many surgeons may not in fact reach true expertise in their career [34].

### Transfer of simulator-trained endovascular skills to real patients

The ultimate purpose of simulators is to positively impact on patient safety through practitioners’ improved performance. Yet trials to prove this are technically and ethically challenging to set up and run. The earliest study to show a clinical skill benefit from medical simulation was conducted in cardiology trainees. Ewy et al. utilized a cardiac simulation mannequin model and showed improved technical ability in fourth-year medical students trained on simulators, examining real patients, compared with their counterparts who had received traditional didactic teaching [35]. This was the first evidence that skills taught on simulators could be transferred into the real clinical world.

Seymour et al. [36] were the first to prove this transferability in a double-blind randomized control trial. Sixteen surgical residents were randomized to receive virtual reality training, or none, and then completed a laparoscopic cholecystectomy on a patient supervised by blinded assessors. Virtual reality trained candidates performed quicker with fewer errors and less non-target tissue damage [36]. There is now evidence of transfer of skills using a colonoscopy [37] and bronchoscopy simulators [38].

Trials to test for the ‘transferability’ of simulation learnt skills into the operating room are ethically challenging to design. With the knowledge that novice learners benefit most from simulation, and that this may improve patient safety, it is hard to justify a trial where some novice operators will receive ‘no-simulation’ training before attempting a procedure on a patient. One solution from Berry et al. [22] was to use a surrogate patient in their trial using virtual reality and porcine simulators. Twelve vascular surgeons with novice endovascular experience were trained to perform an iliac artery stent using either virtual reality simulation, a porcine model, or a combination of both. Performances were scored using a validated tool for assessing technical skill. The authors demonstrated that virtual reality training improved performance scores on the cadaveric model. This is analogous to transfer into a real clinical setting [22].

Simulation should not be a one-off training exercise and should be integrated into the postgraduate training curriculum for endovascular practitioners. Evidence that this supports a sustained improvement in catheter-based skills would be the ideal.

### A model for endovascular training model

A model for endovascular training would have an arterial tree that closely resembles the human body, multiple appropriate branches, antegrade, pulsatile flow, at normal body temperature, multi-layered vessels of normal human arterial calibre and the potential for dissection. It should allow trainees percutaneous needle access and the physical characteristics of limited elasticity found in human arteries, especially those of patients with peripheral artery disease [23]. The realism afforded by such a model could mean a quicker progression to the autonomous stage of learning as described by Fitts, Posner and Kopta in their respective learning theories due to a less steep transitional curve when performing procedures on real patients. Similarly, the variety and realistic tissue feedback that such a model will provide should enable the learner to have sufficient information to fully correlate the relationship between the manoeuver outcome and their control of the movement parameters, a crucial component of motor skill acquisition according to Schmidt’s theory as discussed above.

The model should exhibit face validity; that it is both acceptable and realistic to use. It should also demonstrate construct validity, in measuring the trait that it purports to measure. This is commonly interpreted as the model’s ability to differentiate between practitioners of varying ability [12].

Equally important is that it must be efficacious in its ability to improve a candidate’s performance of endovascular skills when used for training whilst remaining cost-effective.

With regards to the most balanced option for widespread use, one should bear in mind that logistical and regulatory limitations prevent widespread use of cadaveric and animal models. Virtual reality simulation is promising, especially with the option of patient-specific rehearsal but cost remains a prohibitive factor.

## Conclusion

The field of endovascular intervention is rapidly evolving. Many UK trainees seek additional training abroad to gain endovascular competence. Endovascular simulators provide a safe and efficacious platform for trainees to hone their basic skills and break the early part of their learning curve away from patients, thus enhancing patient safety. New advances in technology mean simulators can continue to provide an important training adjunct for even the most experienced practitioners. The biggest current barrier to the routine integration of simulation into endovascular training is the lack of an agreed curriculum. This means that simulators remain a non-essential adjunct and thus are universally underutilized. Concerted efforts in the UK are being made to enhance the role of simulation especially in early stage endovascular training [39].

### Disclosures

No grants or other financial support to declare.
